# Supplementary material for: Out-of-sequence skeletal growth causing oscillatory zoning in arc olivines
Source: Nat Commun. 2021 Jul 1;12:4069. doi: 10.1038/s41467-021-24275-6 (PMC8249515; doi:10.1038/s41467-021-24275-6)
Supplement: Supplementary file 1 — Supplementary Information [file 41467_2021_24275_MOESM1_ESM.pdf]

Supplementary Information

Out-of-sequence skeletal growth causing oscillatory zoning in arc olivines

Pablo Salas[1]*, Philipp Ruprecht[2], Laura Hernández[3], Osvaldo Rabbia[3]

[1]Departamento de Ciencias de la Tierra, Universidad de Concepción, Chile. **E-mail: pabsalas@udec.cl*

[2]Department of Geological Sciences and Engineering, University of Nevada, Reno, United States.

[3]Instituto de Geología Económica Aplicada GEA, Universidad de Concepción, Chile.

Content

	Local setting of Los Hornitos cones………………………………………	3
Fig. S1	Stratigraphic relations and distribution of eruptive products……………	3
Fig. S2a	Histogram of different olivine groups…………………………………...	4
Fig. S2b	Examples of crystal of different groups………………………………...	5
Fig. S3	Exemplary olivine crystal describing a crystal frame……..……………...	6
Fig. S4	Images of immatures dendritic olivines and EPMA data points………..	7
Fig. S5	Compositional profiles along three additional mature olivines ………...	8
Fig. S6a	Orientation parameters in OL_37……………………………………...	9
Fig. S6b	Orientation parameters in OL_50……………………………………...	10
Fig. S6c	Orientation parameters in OL_32……………………………………...	11
Fig. S7	Model of depletion of compatible components under a CBL…………...	12
Fig. S8	Spinel composition………………………………………………………	13
Fig. S9	Time scale calculations…………………………………………………..	14
	Exploratory analysis of olivine zoning by X-ray maps…………………..	15
Fig.S10	X-ray intensity maps of OL_38 and OL_35………………………………	16
	References………………………………………………………………...	17

Local setting of Los Hornitos cones

Los Hornitos are a pair of mafic monogenetic volcanoes emplaced in the Central-South Andes of Chile (35.7°S). These vents are located in the Descabezado Grande Volcanic Field about 15 km SSW of Descabezado Grande volcano.

The cone morphology suggests post-glacial emplacement with the last glacial maximum regionally having been established at ~25 ky (ref. [1]). The preservation state of eruptive products and the stratigraphic superposition of the lava field erupted by the East cone above that of the West cone constrains their relative age relation with the East cone being younger.

For this research we focus on the tephra deposits associated with the initial explosive stage of the younger cone. We exposed a ~2.5 m thick tephra section about 300 m E of the vent area and sampled ash to lapilli size tephra layers. The base of the section was not reach.

Fig. S1

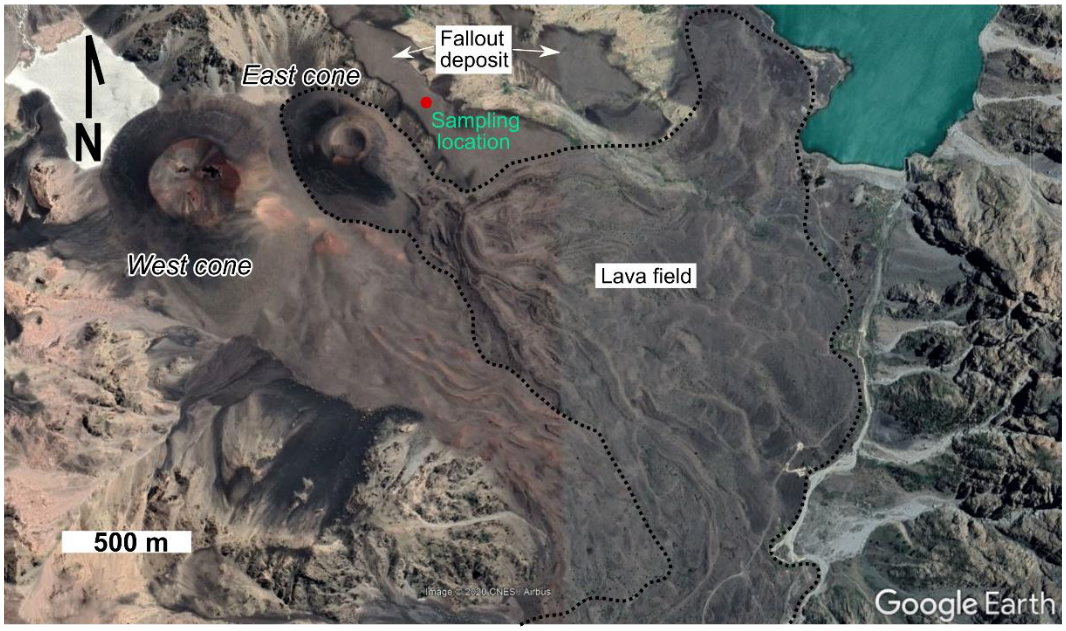

Stratigraphic relations and distribution of eruptive products from the Los Hornitos cones. The segmented line highlights the position of the younger pyroclastic cone and the distribution of the lavas erupted during the late effusive stage. In addition, the initial tephra deposits, are shown with white arrows. Image captured from Google Earth (May 2021; Image © 2021 CNES / Airbus).

Fig. S2a

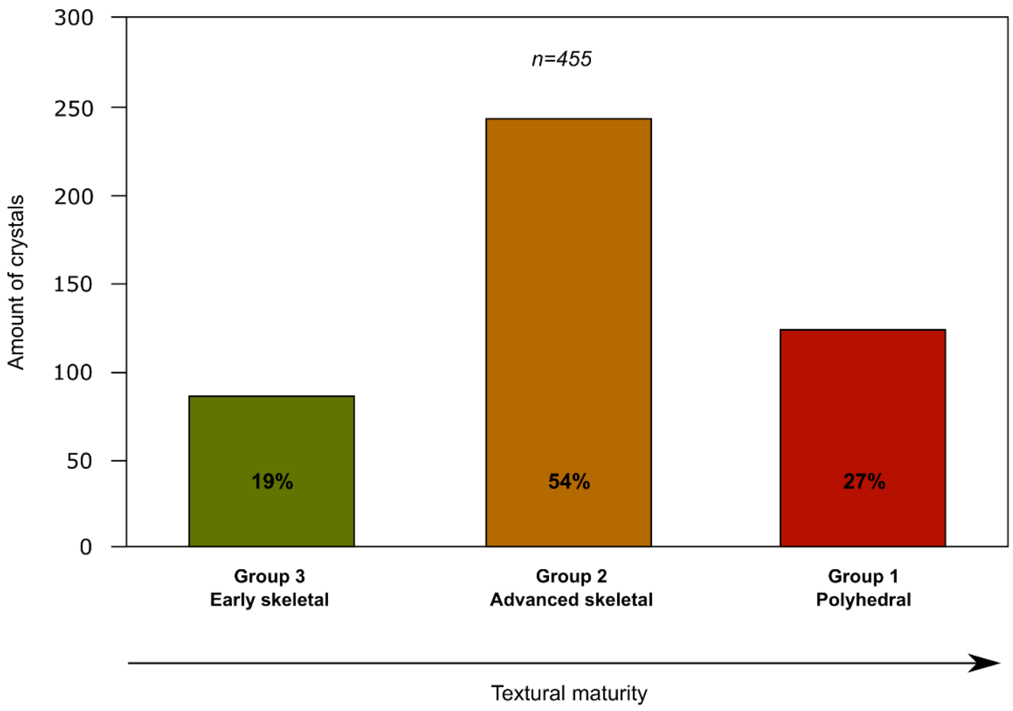

Histogram of modal abundances of groups of Los Hornitos olivine. Groups 1 through 3 of olivine crystals are according to their textural maturation described in the main text and separated into early skeletal, advanced skeletal and polyhedral.

Fig. S2b

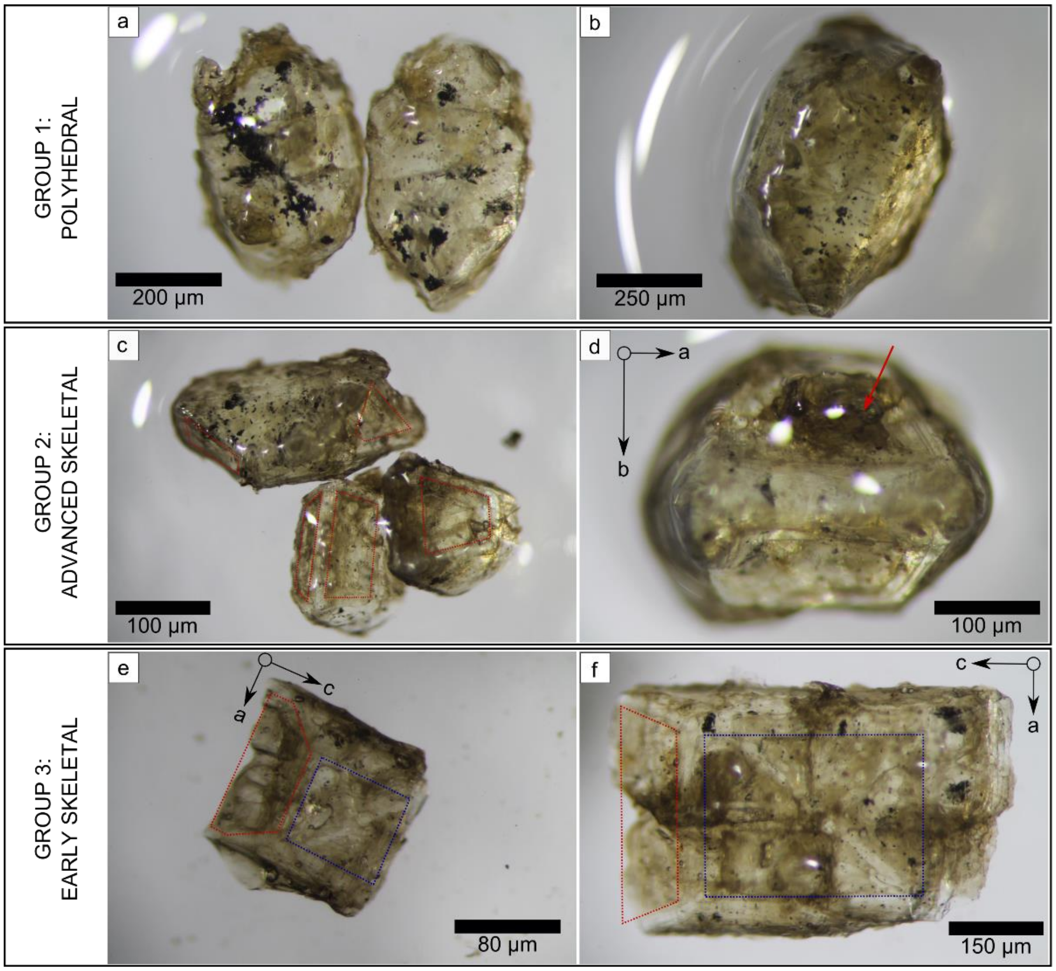

Additional examples of olivine crystals by their textural category observed under the binocular microscrope. a) Two euhedral to subhedral olivines with well-developed external faces. b) Euhedral olivine with a fully polyhedral habit. Note the absence of internal cavities in crystals of a and b. c) Three near-polyhedral olivines retaining skeletal features in the (021) face (segmented red lines). d) Detail of a skeletal crystal oriented normal to c-axis, showing a prominent pocket of glass trapped in the upper cavity (red arrow). These crystals maintain the typical morphology of olivine while leaving open cavities along the (021) planes. e) and f) Two olivines oriented normal to the b-axis showing an externally skeletal texture but retaining dendritic features in the innermost part along plane ac*.* These immature crystals maintain open cavities along the (021) and (010) faces (red and blue segmented lines, respectively), allowing to observe the four internal dendrites that symmetrically radiate from the central core along the ac plane.

Fig. S3

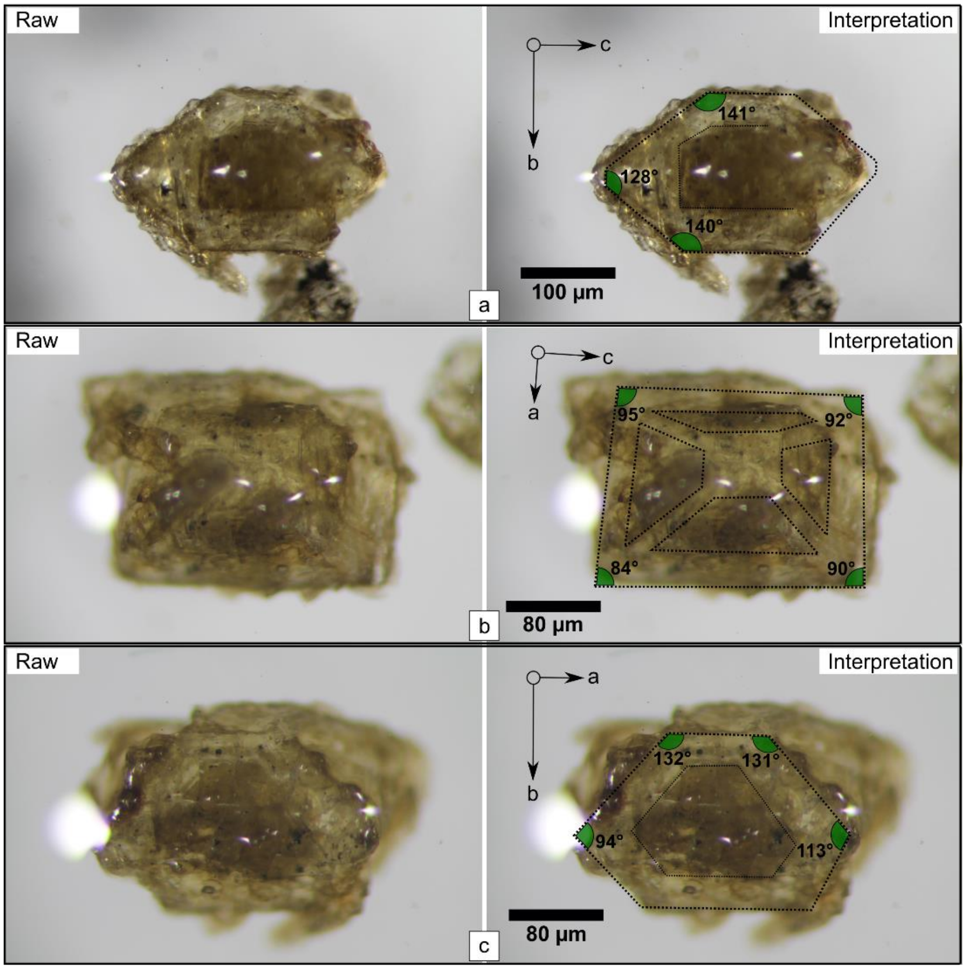

Oriented images of OL-62 showing the early structure of an immature olivine. Left side shows raw images of three different orientations of the same crystal whereas the interpreted crystal morphology, enhanced by segmented lines, is shown on the right side. The interfacial angles for the three positions allow to identify the crystallographic orientation for a) normal to *a*, b) normal to *b* and c) normal to *c*. Note the sharp contacts between the crystal (pale green) with the adjacent glass (greenish brown). See theoretical interfacial angles of olivine in Fig. S6 a, b and c.

Fig. S4

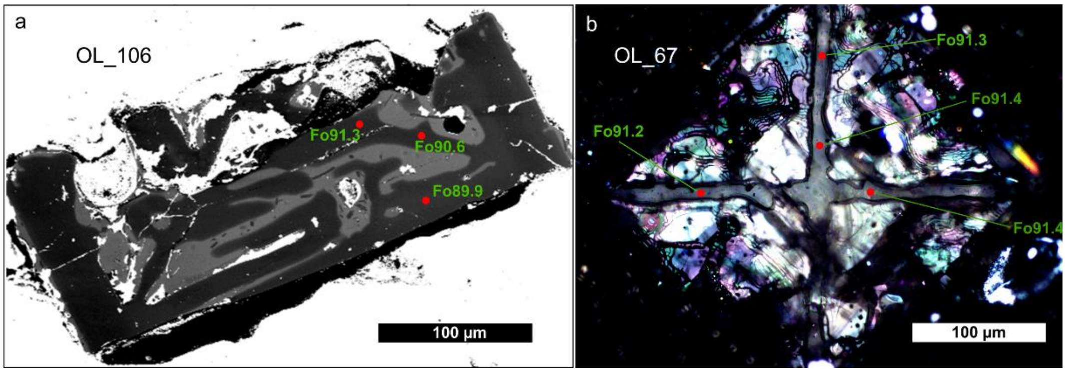

Two immature olivines of group 3 analyzed by EPMA. Note that these crystals are the same as Fig. 1 (a, b) observed under the binocular lenses. Red points indicate the position of EPMA analysis and the respective forsterite composition (Fo). These crystals are texturally equivalent to stage 1b to 2c in Fig. 4. a.- OL_106 observed in a BSE image showing the central structure and the outer frame, where data were collected. b.- OL_67 observed in transmitted light under the microscope showing the early morphology in the center of the crystal (analogous to stage 1b in Fig. 4). Both crystals present a normal forsterite zoning.

Fig. S5

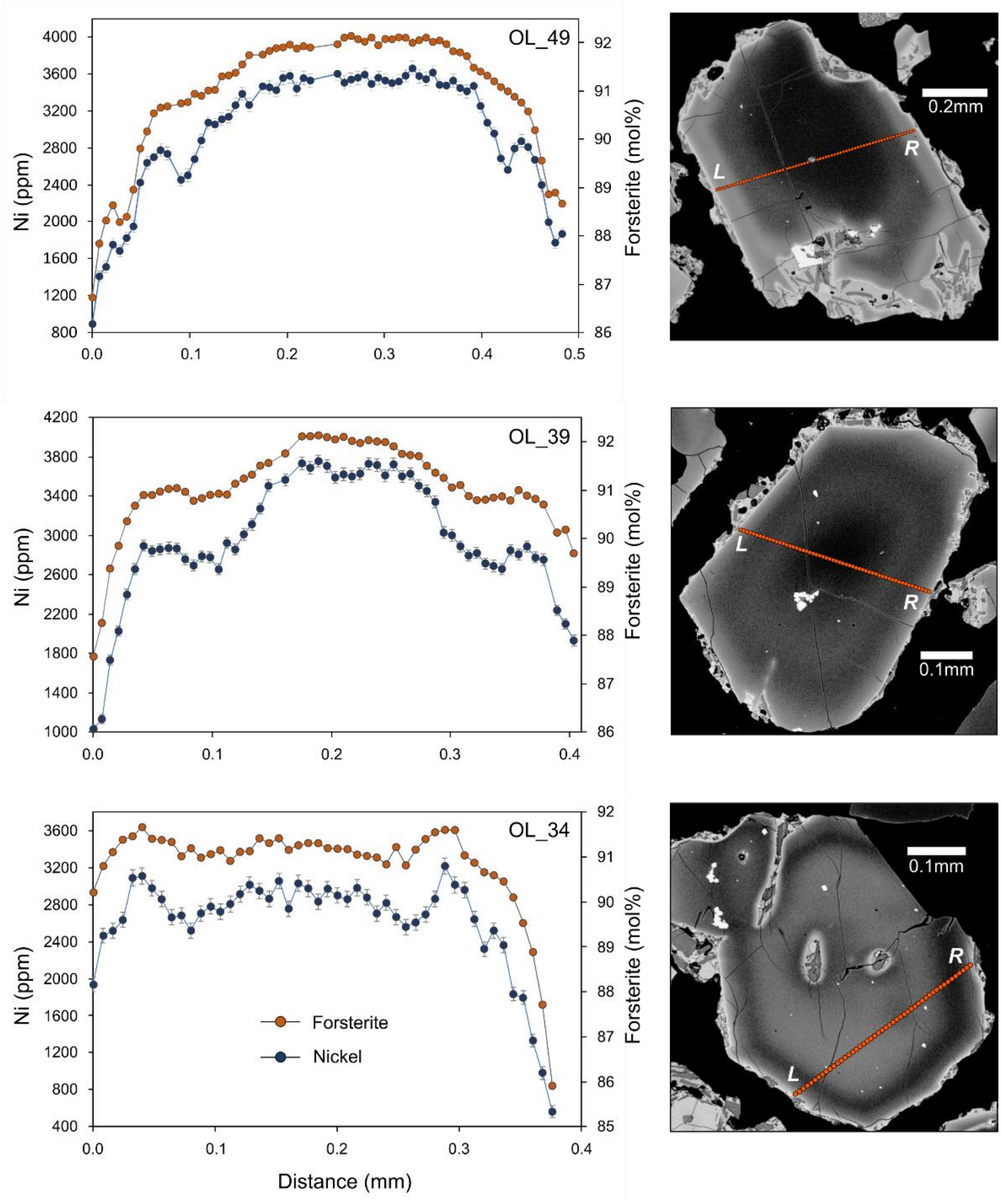

Rim to rim Fo and Ni profiles in three additional examples of mature olivines (OL_49, OL_39 and OL_34) depicting Ni depletions and successive reversals. The absence of a high Fo-Ni inner core in OL_34 indicate that this section does not intersect the center. The crystal orientation is not certain and thus these crystals are not used for diffusion timescale calculations.

Fig. S6a

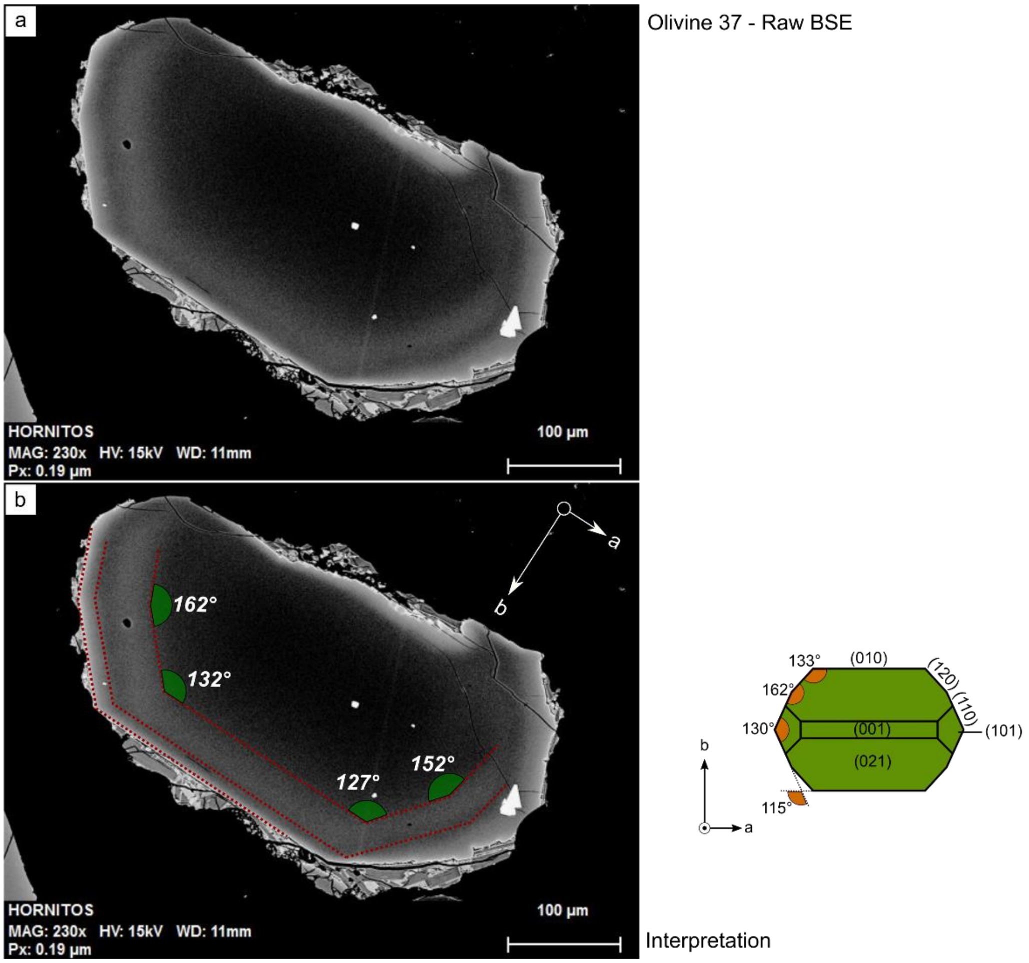

a
Olivine 37 - Raw BSE
HORNITOS
MAG: 230x HV: 15kV WD: 11mm
Px: 0.19 µm
100 µm
b
a
b
162°
132°
127°
152°
HORNITOS
MAG: 230x HV: 15kV WD: 11mm
Px: 0.19 µm
100 µm
133°
(010)
(120)
162°
(110)
130°
(001)
(101)
(021)
115°
b
a
Interpretation

Fig. S6b

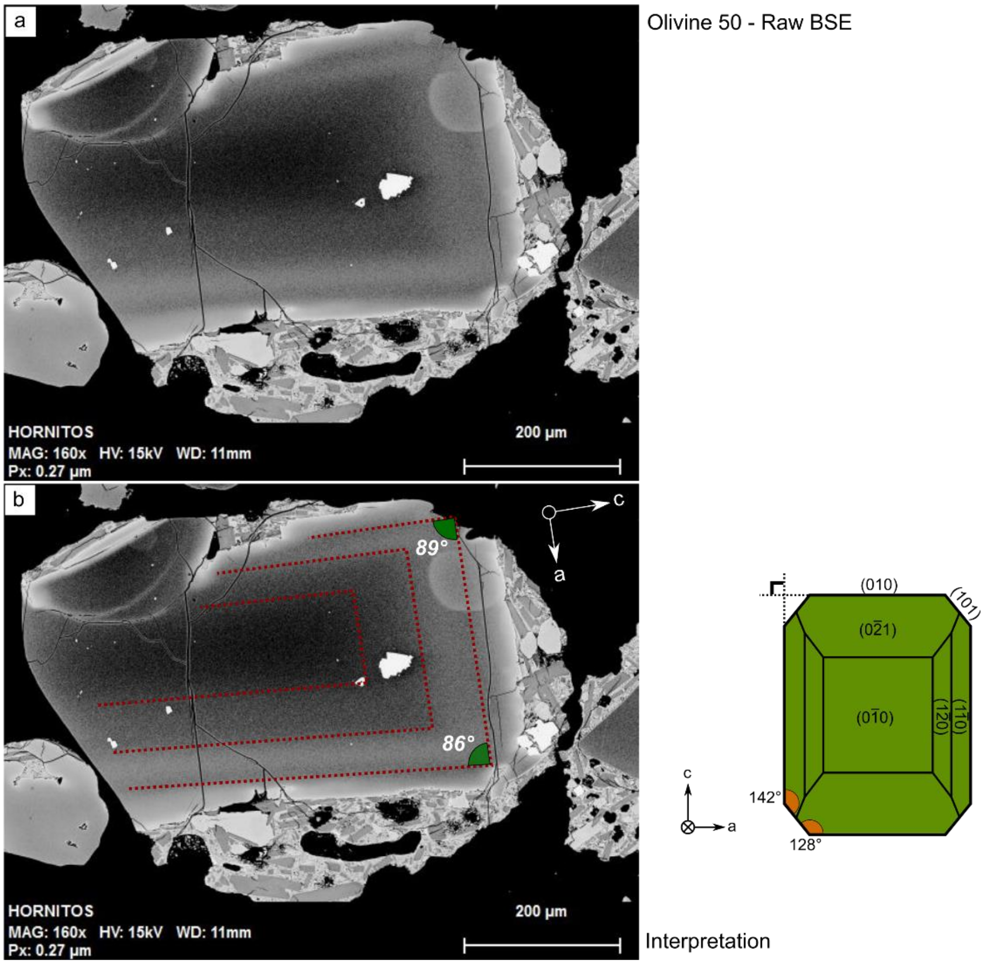

a
Olivine 50 - Raw BSE
HORNITOS
MAG: 160x HV: 15kV WD: 11mm
Px: 0.27 µm
200 µm
b
c
a
89°
86°
HORNITOS
MAG: 160x HV: 15kV WD: 11mm
Px: 0.27 µm
200 µm
(010)
(101)
(02̄1)
(01̄0)
(12̄0)
(11̄0)
c
a
142°
128°
Interpretation

Fig. S6c

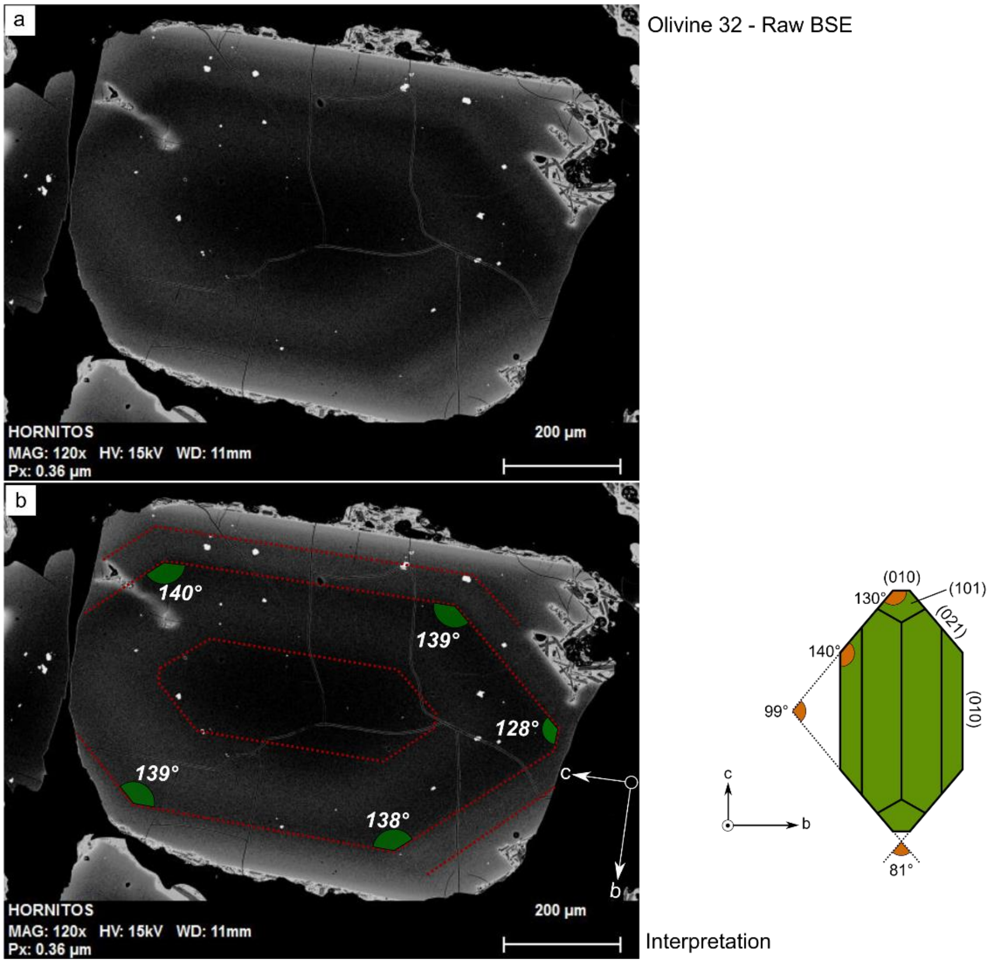

Optical analysis of interfacial angles denoting the crystallographic orientation of the olivine section. a) Olivine 37 closely normal to c-axis, b) Olivine 50 closely normal to b-axis and c) Olivine 32 closely normal to a-axis (mineralogical data taken from Welsch et al[2]).

Fig. S7

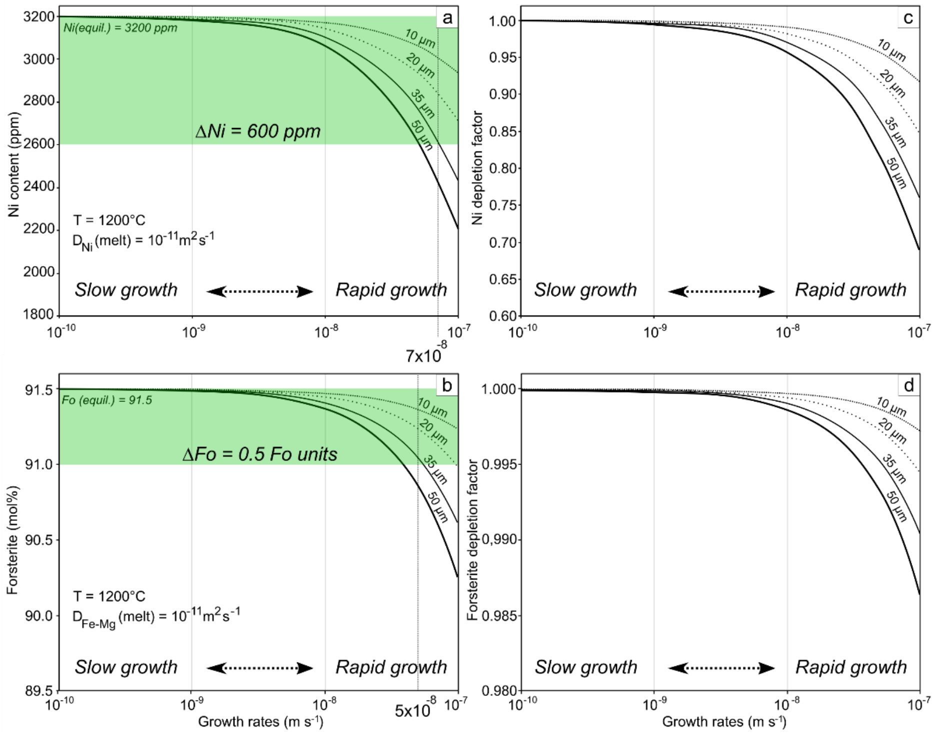

Growth model accounting for boundary layer effects. Modeling the depletion of compatible components using the equation of Watson and Müller[3] for an olivine growing under the effects of a compositional boundary layer (CBL) using different thicknesses (segmented and continuous curves) along with slow to rapid growth rates. Green fields in a and b represent broad compositional ranges observed in natural samples from Los Hornitos. a) Ni and b) Fo variations from an initial equilibrium composition of $Fo_{91.5}$ and 3200 ppm of Ni. Depletion factors of c) Ni and d) Fo, determined as the ratio of the calculated disequilibrium concentration over the initial equilibrium condition, show the considerable larger impact on Ni compared to minor variations in Fo.

As *Ni depletions* in olivine represent growth under a CBL, the thickness of those structures measured in the rim-to-rim profiles indicate the potential extent of the layer. Based on the measuring of the five imaged olivines (Fig. 2 and Fig. S3), we determined the thickness to be between 20 to 50 µm. Using a mean value of 35 µm along with the broad values of ΔFo and ΔNi observed in the depletions of the imaged crystals (see Fig. 2 and Fig. S3), we obtain growth rates on the order of $5x10^{-8}$ to $7x10^{-8}$ ($m\ s^{-1}$) (as shown in Fig.5 a,c).

For calculations in our model we used average distribution coefficients for Mg, Fe ($Kd_{Mg}^{ol\text{-}melt} = 5$, $Kd_{Fe}^{ol\text{-}melt} = 1.65$; e.g., ref. [4]) and Ni ($Kd_{Ni}^{ol\text{-}melt} = 10$; e.g., refs. [4–7]). At 1200°C the diffusivities of Fe and Mg in the melt are of similar magnitude in basaltic melts[8] so we used a common value of $D_{Fe\text{-}Mg}^{melt} = 1 \times 10^{-11}$ (m^2/s) [ref. [8]]. The Ni diffusivity in the melt was approximated to the values reported by Zhang et al.[8] for a haploandesitic melt with values identical to the ones used for Fe and Mg.

Fig. S8

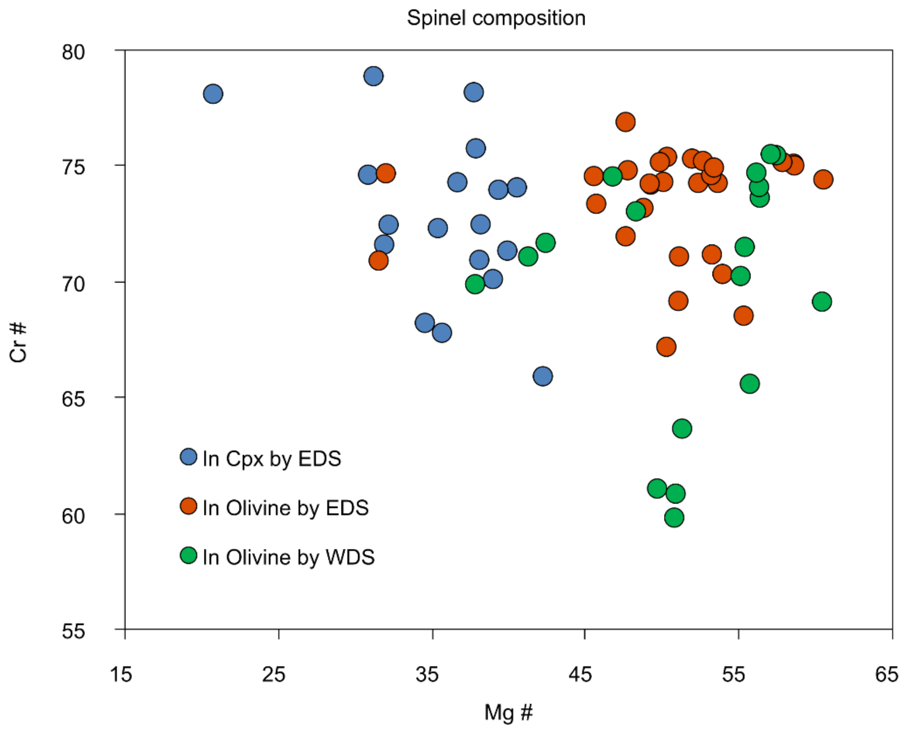

Composition of clinopyroxene and olivine hosted spinels determined by EDS (blue and orange circles) and by WDS (green circles). Most of the olivine hosted spinels plot at Mg# >45. Five exceptions plots at Mg# ≤42 and those are spinels located in the outermost rim of olivine (where Fo ≤~88), in equilibrium with clinopyroxene. By contrast, spinels hosted in clinopyroxene are more iron rich with Mg# <42.

Fig. S9

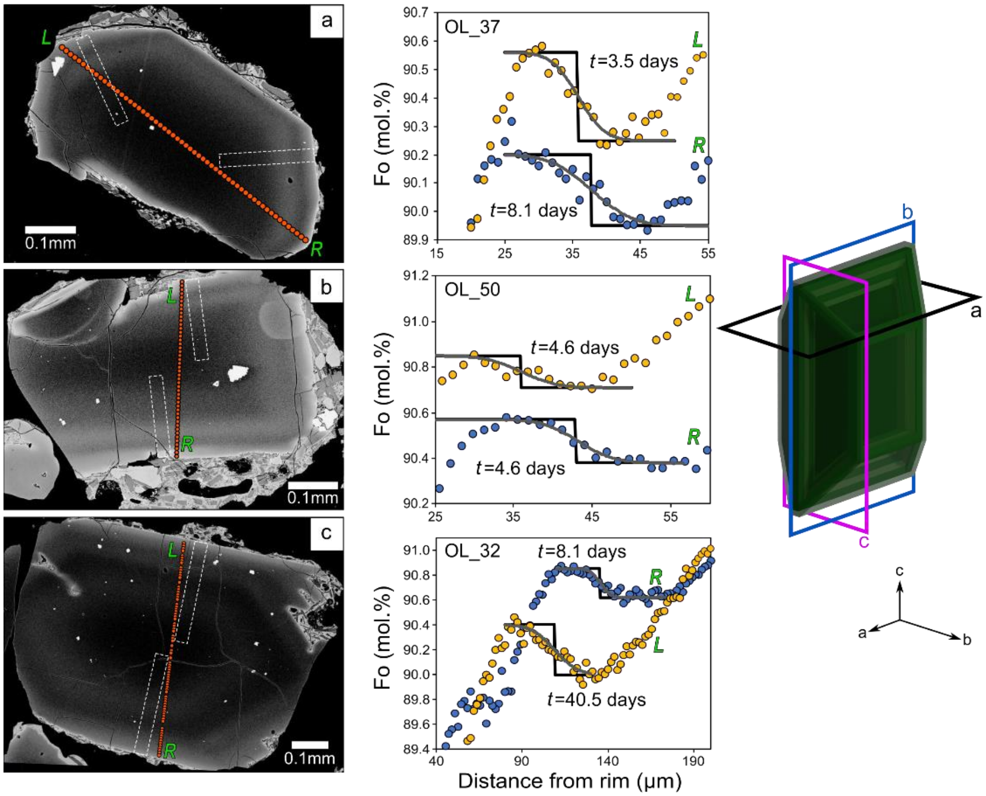

Calculated diffusion timescales from major element zoning. Timescale calculations at 1150°C for three mature olivines with a good control on the orientation of the crystal section. The rim-to-rim transects spot analyses (orange circles) were used to calibrate the grayscale image. The calibrated image was then used to extract traverses of major element zoning (white dashed boxes, see figure 2 for the full profile) to calculate diffusion profiles for timescale estimates. Note, the shown profiles and associated diffusion modeling is restricted to the most pronounced reversals near the rim. a) Olivine 37, b) Olivine 50 and c) Olivine 32 oriented closely normal to the crystallographic axis c, b and a, respectively. Five of the six analyzed traverses provide timescales of <10 days and only one traverse provides a timescale of 40.5 days.

Exploratory analysis of olivine zoning by X-ray maps

Two crystals oriented normal to the axis c were analyzed by X-ray intensity maps. OL_38 consist of an immature olivine from group 3, with a textural maturity comparable to that of stage 2c in Fig. 4. Magnesium map (Fig.S10a) show a normal zoning with the highest distributions occurring in an elongated area at the center of the crystal along axis a. Close to the crystal corner, this area is bounded by a slightly rounded tip (black arrows #1), where lower distribution of magnesium occurs toward the rim. Nickel map (Fig. S10b) reproduce the distribution of Mg, but also show a sharp contact close to the crystal corner (black arrows #2), where a Ni depletion (~20 µm) is consecutively followed by a slight increase in the distribution of Ni toward the rim. The shape of the area depleted in Ni reproduce the external faces of the crystal. Interestingly, the distribution of P (Fig. S10c) is even more complex but show that the highest distribution occurs right in the area occupied by the Ni depletion (black arrows #3). In addition, P map also shows a delicate distribution constituted by three elongated bands (~20 microns thick; coarser arrows) along axis a, and those are separated by areas of low or no distribution of P.

On the other hand, OL_35 consist of a mature olivine retaining a skeletal texture in the (021) face (Fig. S10 d, e, f). This crystal is irregularly broken along plane (010). Magnesium map (Fig. S10d) shows normal distribution but preserving an oscillatory pattern towards the right corner, where focused maps of Ni and P were acquired. The inner high distribution of Mg occupies an elongated band of ~200 x 100 µm along axis a, limited by a rounded tip that broadly mimic the shape of external faces. Here the distribution of Mg drastically drops in a ~30 microns thick band (arrows #4) and is followed by a new band of high Mg (~40 microns thick). This area is limited by a sharp contact with a ~10 µm band of lower distribution of Mg (arrows #5) that follows the external shape of the crystal, and successively another band of slightly higher distribution of Mg toward the rim. Nickel distribution map (Fig. S10e) follow the pattern depicted by Mg, overlapping the more remarkable zones of high and low distributions but with significant less contrast (arrows #6 and #7). Finally, the distribution of P (Fig. S10f) occurs as two well defined lines that follow the external contour of the crystal. The inner P-line (arrows #8) distributes towards the external border of the outer Ni depletion (better observed as Mg depletion), whereas the outer P-line occurs in the internal border of the outer Mg-rich band near the rim (arrow #9).

For both crystals, note the remarkable affinity in the distribution and morphology of major (Mg), minor (Ni) and trace (P) elements, thus confirming a common process operating during the development of these chemical features.

Fig. S10

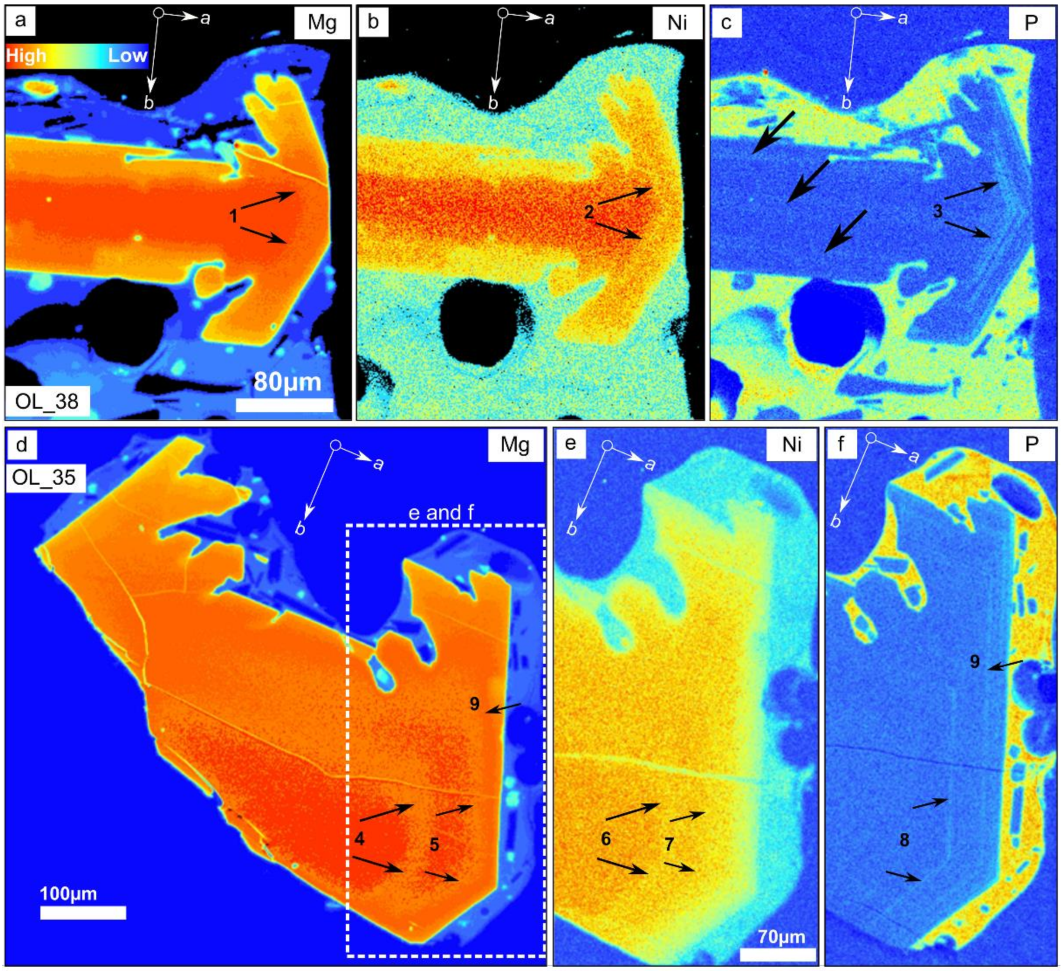

X-ray intensity maps of two olivines of different textural maturity. a, b, c corresponds to Mg, Ni and P distribution maps in OL_38, respectively. d, e, f are the distribution maps of Mg, Ni and P of OL_35.

References

1. Singer, B., Hildreth, W. & Vincze, Y. ^{40} Ar/ ^{39} Ar evidence for early deglaciation of the central Chilean Andes. *Geophys. Res. Lett.* **27**, 1663–1666 (2000).
2. Welsch, B., Faure, F., Famin, V., Baronnet, A. & Bachèlery, P. Dendritic Crystallization: A Single Process for all the Textures of Olivine in Basalts? *J. Petrol.* **54**, 539–574 (2013).
3. Watson, E. B. & Müller, T. Non-equilibrium isotopic and elemental fractionation during diffusion-controlled crystal growth under static and dynamic conditions. *Chem. Geol.* **267**, 111–124 (2009).
4. Beattie, P., Ford, C. & Russell, D. Partition coefficients for olivine-melt and orthopyroxene-melt systems. *Contrib. Mineral. Petrol.* **109**, 212–224 (1991).
5. Hart, S. R. & Davis, K. E. Nickel partitioning between olivine and silicate melt. *Earth Planet. Sci. Lett.* **40**, 203–219 (1978).
6. Kinzler, R. J., Grove, T. L. & Recca, S. I. An experimental study on the effect of temperature and melt composition on the partitioning of nickel between olivine and silicate melt. *Geochim. Cosmochim. Acta* **54**, 1255–1265 (1990).
7. Straub, S. M., LaGatta, A. B., Martin-Del Pozzo, A. L. & Langmuir, C. H. Evidence from high-Ni olivines for a hybridized peridotite/pyroxenite source for orogenic andesites from the central Mexican Volcanic Belt: Andesite petrogenesis in Central MVB. *Geochem. Geophys. Geosystems* **9**, (2008).
8. Zhang, Y., Ni, H. & Chen, Y. Diffusion Data in Silicate Melts. *Rev. Mineral. Geochem.* **72**, 311–408 (2010).
