# Supplementary material for: Out-of-sequence skeletal growth causing oscillatory zoning in arc olivines
Source: Nat Commun. 2021 Jul 1;12:4069. doi: 10.1038/s41467-021-24275-6 (PMC8249515; doi:10.1038/s41467-021-24275-6)
Supplement: Supplementary file 3 — Description of Additional Supplementary Files [file 41467_2021_24275_MOESM3_ESM.pdf]

Description of Additional Supplementary Files

File Name: Supplementary Data 1

Description: Electronic file containing the compositional data of olivine discussed through the text se parated by groups.

File Name: Supplementary Data 2

Description: Electronic file containing the compositional data of clinopyroxene and glass used during thermobarometry calculations.

File Name: Supplementary Data 3

Description: Electronic file containing the compositional data of spinels measured by EDS and WDS.

File Name: Supplementary Data 4

Description: Summary of thermobarometry results.

File Name: Supplementary Data 5

Description: Compositional data on H_2O, S, Cl, P and F measured by SIMS in external glasses surro unding olivine crystals.
